# More than a communication disorder: inequities in the financial toxicity of post-stroke aphasia

**DOI:** 10.3389/fstro.2024.1507025

**Published:** 2025-01-07

**Authors:** Molly Jacobs, Charles Ellis

**Affiliations:** ^1^Department of Health Services Research, Management and Policy, College of Public Health and Health Professions, University of Florida, Gainesville, FL, United States; ^2^Department of Speech, Language and Hearing Sciences, Communication Equity and Outcomes Laboratory, College of Public Health and Health Professions, University of Florida, Gainesville, FL, United States

**Keywords:** stroke, aphasia, financial toxicity, disparities, income

## Abstract

**Introduction:**

Aphasia, a communication disorder often resulting from stroke, can have profound impacts on both health outcomes and financial wellbeing. While the physical and cognitive consequences of stroke are well documented, the financial strain, or “financial toxicity,” associated with managing chronic conditions like aphasia remains underexplored. Furthermore, financial toxicity is not experienced equally across racial and ethnic groups, with disparities driven by socioeconomic factors, access to healthcare, and structural inequities. This study compares the financial toxicity of people with aphasia (PWA) to those with stroke alone, examining differences across racial and ethnic groups to highlight disparities in economic burden.

**Methods:**

This study utilized data from the Medical Expenditure Panel Survey (MEPS) collected between 2018 and 2021 to examine the financial toxicity of PWA compared to those with stroke only. Financial toxicity was assessed using self-reported income and wealth data from the MEPS. Individual-level income and wealth values were calculated from the self-reported financial data to quantify the financial burden. Fixed effects regression models were employed to account for unobserved individual heterogeneity, controlling for time-invariant characteristics. Interaction terms were included in the models to capture the differential financial impacts of aphasia on Black and Hispanic individuals, compared to other racial and ethnic groups. The analysis examined both within-group and between-group differences in financial toxicity, highlighting potential racial and ethnic disparities among those affected by aphasia.

**Results:**

Approximately 18.71% (*N* = 281) of respondents who reported having a stroke also had aphasia. After controlling for demographic, health, and household characteristics, PWA had 21% lower income and 7% lower wealth compared to stroke survivors without aphasia. Aphasia had a disparate impact on the income (−29%) and wealth (−24%) of Black stroke survivors. These findings were consistent across different model specifications, highlighting the robustness of the results indicating racial inequity in the financial toxicity of post-stroke aphasia.

**Conclusion:**

This study showed the financial impact of post-stroke aphasia and the disparate burden among Black PWA. The findings highlight the need to address the financial ramifications of post-stroke morbidities such as aphasia among vulnerable populations.

## Introduction

Aphasia is a communication disorder characterized by impairment of language that can negatively impact both speech and comprehension abilities as well as the ability to read or write (National Aphasia Association, 2024a). According to the National Aphasia Association, ~2 million Americans have aphasia and ~180,000 individuals acquire the communication disorder each year (National Aphasia Association, 2024b). Even in its mildest form, aphasia can cause significant disruption in communication ability (Armstrong et al., 2013). People with aphasia (PWA) have an increased risk of depression, fatigue, anxiety, and insomnia when compared to stroke survivors without the condition (Hersh et al., 2024). PWA also report loneliness (Hersh et al., 2024), loss of friendships (Archer et al., 2024; Madden et al., 2023), disruptions and dissatisfaction with healthcare experiences (Carragher et al., 2024; Tomkins et al., 2013), reduced social participation (Quique et al., 2023), and reductions in their quality of life (Filipska-Blejder et al., 2023).

Although substantial research related to the communication challenges of PWA (Poirier et al., 2024) and their loved ones (McGurk and Kneebone, 2013) exists, there has been virtually no research examining the financial problems that PWA face and the additive burden of the condition. Although recent estimates suggest that the annual cost of aphasia is $15.8 billion annually, or $30,599.78 per PWA, as related to medical/healthcare expenditures, lost wages, and informal caregiving (Jacobs and Ellis, 2023), virtually nothing is known about the specific factors that contribute to the “financial toxicity” of having aphasia. Financial toxicity refers to the “toxic effects” that a health condition can have on the financial resources of individuals with a health condition (Pisu and Martin, 2022). Financial toxicity is the result of the cumulative expenses associated with receiving medical care. Estimates suggest that 56% of US adults experience financial toxicity and have unavoidable medical expenses, even though many have insurance coverage (Yabroff et al., 2019).

Financial toxicity among PWA is potentially a devastating problem because of the nature of the communication disorder and the impact that it has on the PWA who are also recovering from stroke. For example, stroke itself is a condition that frequently requires long-term, organized, and high-cost care (Sarzyńska-Długosz, 2023). Communication issues such as aphasia that arise post-stroke are frequently one of the multiple potential problems that stroke survivors experience. Yet the burden is greater among individuals with communication issues because of the greater likelihood of disrupted healthcare access, utilization, and satisfaction over and above the experiences of stroke survivors without aphasia (Cummings, 2023). Some PWA are left with unmet needs that are linked to poor patient–provider communication due to aphasia (Anemaat et al., 2024). Therefore, understanding the issue of financial toxicity among stroke subpopulations (i.e., those with aphasia) is critically important to the determination of the multiple factors that drive stroke- and aphasia-related outcomes and the need for tailored interventions designed to optimize these outcomes. This study was designed to explore financial toxicity among PWA to profile financial toxicity among PWA and determine the sociodemographic factors that contribute to greater financial toxicity in this population compared to those individuals without aphasia.

## Materials and methods

### Data

This panel data study used longitudinal data from the Medical Expenditure Panel Study (MEPS) collected between 2018 and 2021 (Agency for Healthcare Research Quality, 2023). The sample was restricted to adults aged 18 years and above. The MEPS data provide nationally representative estimates of healthcare use, expenditures, sources of payment, and health insurance coverage for the US civilian non-institutionalized population (Agency for Healthcare Research Quality, 2023). The MEPS Household Component (HC) also provides estimates of respondents' health status, demographic and socioeconomic characteristics, employment, access to care, and satisfaction with healthcare. The panel design of the survey includes rounds of interviews covering four full calendar years, allowing for analysis of person-level changes in selected variables such as expenditures, health insurance coverage, and health status.

The Panel 23 Four-Year Longitudinal Data File from the MEPS Household Component (MEPS-HC) (Agency For Healthcare Research Quality, 2012) provided data collected on a nationally representative sample of the civilian non-institutionalized population of the United States for the 4-year period 2018–2021. This dataset includes records for 7,080 persons in Panel 23 who were respondents during the period they were in-scope for the survey (i.e., part of the civilian non-institutionalized population) over the 4-year period. Only persons with positive person-level weights (PERWT18F, PERWT19F, PERWT20F, or PERWT21F) are included in the longitudinal Public Use Files (PUF) data. Data are available for all nine rounds for 87.46% of the cases (6,192 persons). The remaining 12.54% (888 persons) did not have data for one or more rounds but were in-scope for all rounds in which they participated in the survey. These persons are those who were born, died, were in the military or an institution, or who left the country during the 4-year period. In contrast, persons in the panel who participated in the survey for only part of the period they were in-scope are not included in this file. Variables in the file pertaining to survey administration, demographics, employment, health status, disability days, quality of care, patient satisfaction, health insurance, and medical care use and expenditures were obtained from the MEPS 2018, 2019, 2020, and 2021 Full-Year Consolidated Files (HC-209, HC-216, HC-224, and HC-233, respectively).

### Sample

In each year of the panel, respondents indicated whether they had ever been told by a doctor or other health professionals that they had a stroke. To identify those with post-stroke aphasia, the subset of individuals who reported a prior stroke diagnosis in the Panel 23 Four-Year Longitudinal Data File were merged with the 2018 (HC-231), 2019 (HC-222), 2020 (HC-214), and 2021 (HC-207) MEPS Medical Condition files using individual-specific surrogate identifiers. These files contain household-reported medical conditions from a nationally representative sample in the calendar year. Each record represents one current medical condition reported during the respective data collection period. Reported conditions were reported by interviewers using a condition picklist with the International Classification of Diseases, Medical Classification, 10th Revision (ICD-10-CM) codes already assigned to conditions in the list. To preserve confidentiality, all the conditions in the file were grouped into three-digit diagnosis code categories, rather than using the fully specified ICD-10-CM codes. Codes R47 and I69 indicated a stroke survivor with aphasia.

This study was granted exempt status by the University of Florida Institutional Review Board (IRB) as it does not involve human subjects. Similarly, informed consent was not obtained because the study involved the secondary analysis of existing data.

### Financial outcomes

Two financial indicators were created—income and wealth—consistent with the methodology used by Bernard et al. (2009). Conceptually, income was considered the money an individual earns in a calendar year, while wealth was the value of a person's assets minus their debts. Income was calculated as money received during the current calendar year from Individual Retirement Accounts (IRAs), Keogh Accounts, or 401Ks; private pensions, military retirement, other federal government employee pensions, state or local government employee pensions, or annuities; Social Security and equivalent tier 1 Railroad Retirement benefits; wages or salary, tips, commissions, or bonuses; business or practice; unemployment compensation; veterans payments; workers compensation; Supplemental Security Income (SSI); and public assistance payments. Wealth included net income as well as the total net gain or loss from alimony; dividends; cash contributions received from non-household members such as gifts or sporadic payments; interest income from savings accounts, bonds, NOW accounts, money market accounts, or similar types of investments; the sale of property or other assets including home(s); estates, trusts, partnerships, S corporations, rent, and royalties; and other sources specified elsewhere.

### Individual characteristics

The variables used in this analysis are described below. For categorical variables, “^*^” denotes the reference group.

Individuals' characteristics included sex (male^*^, female), region of geographic residence (Northeast^*^, Midwest, South, West), age in years (<65^*^, ≥65), household size (1–2^*^, ≥3), education (less than high school/high school, beyond a high school education^*^), Black race (Black, non-Black^*^), and Hispanic ethnicity (Hispanic, non-Hispanic^*^). Race and ethnicity were self-reported by respondents. MEPS respondents provided information about their insurance status and type of coverage, which was then categorized into two groups: private health insurance and non-private health insurance^*^.

An indicator for employment status was created using two survey items. First, respondents reported weekly hours worked at their current main job (CMJ) in each round. Those reporting working more than zero hours at their CMJ were considered employed. To validate this classification schema, responses were compared to reported reasons for not working in the same round. This comparison ensured that indicators of employment captured financially compensated market work rather than part-time student or retirement jobs.

Comorbid disease burden was captured using the Grouped Charlson Comorbidity Index (CCI), which has been used frequently in studies involving the MEPS data (de Groot et al., 2004; Salami et al., 2018; Charlson et al., 1987). For this analysis, however, the CCI excluded stroke to avoid collinearity in regression analyses. Two categories of the CCI included zero or one comorbidity^*^ and two or more comorbidities. Finally, to account for differences in healthcare utilization and spending, total healthcare expenditure—defined as the sum of direct payments for care provided during the year, including out-of-pocket payments and payments by private insurance, Medicaid, Medicare, and other sources—was included as a dichotomous variable indicating an annual healthcare expenditure in the top 30% of the annual distribution. This variable was referred to as “high healthcare costs.”

### Statistical analysis

This analysis evaluated the association between income, wealth, and aphasia among stroke survivors accounting for the differential financial implications of age, employment, and health insurance. This approach integrates the distinct financial mechanisms and safety nets, such as retirement income or disability benefits, that shape the experience of financial toxicity following a stroke. Wealth and income were adjusted for inflation to 2024 dollars and natural-logged because of their skewed distribution, and separate regression models were specified to avoid concerns of collinearity and heteroskedasticity (Equation 1).


(1) $$\begin{array}{l} Outcome_{it}=\beta_{0}+\beta_{1}{Age\geq 65}_{it}+\beta_{2}Female_{it}+\beta_{3}CCI_{it}\\ \;+\beta_{4}BlackRace_{it}+\beta_{5}HispanicEthnicity_{it}\\ \;+\beta_{6}Working_{it}+{\beta_{7}Region}_{it}\\ \;+\beta_{8}Education_{it}+\beta_{9}HighHealthcareCost_{it}\\ \;+\beta_{10}Household\;Size_{it}+\beta_{11}Private\;Insurance_{it}\\ \;+\beta_{12}Aphasia_{it}+{\beta_{13}BlackRace}_{it}\times Aphasia_{it}\\ \;+\beta_{14}HispanicEthnicity\times Aphasia_{it}+\mu_{i}+\tau_{t}+\varepsilon_{it} \end{array}$$

*Outcome*_*it*_ was the outcome variable (income or wealth) in the logarithmic form to be estimated of individual *i* in year *t*. *Aphasia*_*it*_ was the primary explanatory variable assuming a value of 1 for diagnosis of aphasia and 0 otherwise^*^. Residential sampling units and year-fixed effects were denoted by μ_*i*_ and τ_*t*_, and ε_*it*_ is the error term. The remaining covariates indicated characteristics of individual *i* in year *t*. As suggested by the MEPS, all analyses utilized survey-specific commands. These commands allowed for the incorporation of survey weights, the inclusion of multiple waves, and the identification of survey-specific parameters that account for the complex survey design and sampling framework. Using the survey parameters and sampling weights correctly estimated standard errors and generated findings that reflect the national population.

First, descriptive statistics for the “aphasia” and “no aphasia” samples were calculated. The *F*-tests tested for statistically significant differences in characteristics between groups. Next, the fixed effects (FE) regression models evaluated the association between income/wealth (modeled separately), aphasia diagnosis, and individual characteristics. The FE regression modeled individual-level income/wealth changes throughout the panel while controlling for unobserved time-invariant individual heterogeneity that remained constant within the study period. This approach has been used extensively in similar studies and is regarded as statistically robust and conservative (Babiarz and Yilmazer, 2017; Jeon and Pohl, 2017; Gaspar et al., 2021). The results can be interpreted as the relationship between annual changes in income/wealth and the regressors. Significant interaction terms between Black race and Hispanic ethnicity and aphasia indicated between group differences in the association between income/wealth and race/ethnicity.

To test the robustness of the FE analyses, two-part “Heckman-style” models were estimated. The two-part specification accounted for the skewed nature of income and wealth distributions. Two-part models are frequently used when estimating earnings functions to account for the portion of the sample who have no earnings within a given period. In the first stage, a probit model estimated the probability that the outcome was greater than zero conditional on covariates. For those observations with non-zero outcome values, the second stage estimated the outcome conditional on the covariates.

## Results

### Sample characteristics

Table 1 describes the characteristics of both PWA and those without aphasia. Approximately 35% of the PWA were below the age of 65 compared to 49% of those without aphasia (*F* = 12.11, *p* < 0.0001). Nearly 70% of PWA were male compared to only 48% of those without aphasia (*F* = 30.28, *p* < 0.0001). Over 90% of PWA had two or more comorbidities compared to only 80% of those without aphasia (*F* = 15.52, *p* < 0.0001). More than 25% of PWA were Black and 23% were Hispanic compared to only 19% and 13% of those without aphasia (Black *F* = 5.11, *p* = 0.02; Hispanic *F* = 165, *p* = 0.20). Nearly 60% of PWA were employed and 32% of PWA had private health insurance compared to 55% and 34%, respectively, of those without aphasia (working *F* = 63.61, *p* < 0.00021; private insurance *F* = 29.66, *p* < 0.0001). Among PWA, 19% lived with three or more individuals and 23% resided in the South (of those without aphasia 22% resided with 3+ individuals, *F* = 64.25, *p* < 0.0001; 22% resided in the South, *F* = 8.28, *p* = 0.00). Only 6% of PWA had education beyond a high school degree compared to 40% of those without aphasia (*F* = 82.00, *p* < 0.0001). However, 40% of those without aphasia had high healthcare costs compared to 31% of PWA (*F* = 4.53, *p* = 0.03).

**Table 1:** Sample characteristics.

	***N*** = **281, 18.71%**		***N*** = **1,221, 81.29%**		***F*-statistic**	***p*-value**
	**Mean**	**SD**	**Mean**	**SD**		
Income ($0–$56,538.18)	2,337.11	7,179.99	20,916.58	22,405.62	−1.73	0.08
Wealth ($0–$61,458.18)	2,496.82	7,667.64	22,703.74	25,907.05	0.94	0.33
Age < 65	99	35.23	669	48.62	12.11	< 0.0001
Age ≥65	182	64.77	707	51.38		
Male	196	69.01	660	47.97	30.28	< 0.0001
Female	88	30.99	716	52.03		
0–1 comorbidities	22	7.75	237	17.22	15.52	< 0.0001
2+ comorbidities	262	92.25	1,139	82.78		
Non-Black race	208	73.24	1,108	80.52	5.44	0.02
Black race	76	26.76	268	19.48		
Non-Hispanic	220	77.46	1,204	87.5	1.65	0.20
Hispanic	64	22.54	172	12.5		
Not working	116	40.21	624	45.35	63.64	< 0.0001
Working	168	59.79	752	54.65		
Other insurance	190	67.86	771	66.41	29.66	< 0.0001
Private insurance	90	32.14	390	33.59		
Resides with 0–2 people	227	81.07	907	78.12	64.25	< 0.0001
Resides with 3+ people	53	18.93	254	21.88		
Northeast	48	17.14	171	14.73	8.28	0.00
Midwest	28	10	227	19.55		
South	139	49.64	509	43.84		
West	65	23.21	254	21.88		
High school degree or below	132	94.29	820	59.59	482.00	< 0.0001
Education beyond high school	8	5.71	556	40.41		
Healthcare costs < $8,000	197	69.37	837	60.83	4.53	0.03
Healthcare costs ≥$8,000	87	30.63	539	39.17		

### Regression results

Table 2 shows the FE regression results for income and wealth for all stroke survivors (for PWA and those without aphasia). The results indicated that, relative to men, women have 24% (SE = 0.07) lower income and 24% (SE = 0.01) lower wealth. Relative to their counterparts, respondents with educational attainment of a high school degree or beyond (income 45%, SE = 0.08; wealth 46%, SE = 0.09), private insurance (income 33%, SE = 0.07; wealth 32%, SE = 0.08), and who were employed (income 32%, SE = 0.06; wealth 40%, SE = 0.07) had higher income and wealth. Compared to non-Black individuals, Black PWA had significantly lower income (−22%, SE = 0.11) and wealth (−28%, SE = 0.13). Similarly, compared to non-Hispanic individuals, Hispanic individuals had lower income (−48%, SE = 0.12) and wealth (−49%, SE = 0.15). The presence of aphasia was associated with 21% (SE = 0.24) lower income and 7% (SE = 0.29) lower wealth compared to stroke survivors without aphasia. Compared to Black stroke survivors without aphasia, Black PWA had 29% (SE = 0.74) lower income and 24% (SE = 0.90) lower wealth even after controlling for age, employment, educational, and residential heterogeneity.

**Table 2:** Income and wealth fixed effect regression results.

	**Dependent variable: income**				**Dependent variable: wealth**			
		**SE**	***t* value**	**Pr > |*t*|**		**SE**	***t* value**	**Pr > |*t*|**
Intercept	**9.52**	0.16	58.49	< 0.0001	**9.35**	0.19	48.81	< 0.0001
Age ≥65	0.07	0.07	0.99	0.32	**0.21**	0.08	2.65	0.01
Female	**−0.24**	0.07	−3.26	0.00	**−0.24**	0.09	−2.66	0.01
2+ comorbidities	0.08	0.10	0.84	0.40	0.17	0.11	1.48	0.14
Black race	**−0.22**	0.11	−2.11	0.04	**−0.28**	0.13	−2.22	0.03
Hispanic ethnicity	**−0.48**	0.12	−3.91	0.00	**−0.49**	0.15	−3.35	0.00
Currently working	**0.32**	0.06	5.07	< 0.0001	**0.40**	0.07	5.44	< 0.0001
Private health insurance	**0.33**	0.07	5.03	< 0.0001	**0.32**	0.08	4.1	< 0.0001
Resides with ≥3 people	−0.03	0.03	−1.17	0.24	−0.02	0.03	−0.69	0.49
Midwest	−0.02	0.12	−0.17	0.87	−0.05	0.15	−0.31	0.76
South	−0.06	0.11	−0.57	0.57	−0.12	0.13	−0.9	0.37
West	−0.07	0.12	−0.56	0.58	−0.19	0.15	−1.27	0.20
Education beyond high school	**0.45**	0.08	5.73	< 0.0001	**0.46**	0.09	4.83	< 0.0001
High cost of healthcare	−0.03	0.04	−0.65	0.51	−0.04	0.05	−0.87	0.38
Aphasia	**−0.21**	0.24	−2.88	0.04	**−0.07**	0.29	−2.22	0.03
Aphasia^*^Black race	**−0.29**	0.74	−2.39	0.05	**−0.24**	0.90	−2.26	0.04
Aphasia^*^Hispanic ethnicity	0.01	0.78	0.01	0.99	−0.07	0.94	−0.07	0.94

Reference group: Age (< 65), Sex (male), Comorbidities (0–1 comorbidities), Race (non-Black), Ethnicity (non-Hispanic), Household size (resides with 0–2 people), Region (Northeast), Education (high school degree or below), Healthcare cost (lower healthcare costs), Aphasia (no aphasia diagnosis).

Bold indicates significance at a 95% confidence interval.

### Robustness test

Table 3 shows the regression results for the two-stage estimation models for income and wealth. The results mirror the findings discussed above. On average, Black stroke survivors had 26% (95% CI = −0.42, −0.10) lower income and 34% (95% CI = −0.54, −0.15) lower wealth compared to non-Black stroke survivors and Hispanic stroke survivors had 55% (95% CI = −0.81, −0.30) lower income and 53% (95% CI = −0.81, −0.25) lower wealth than non-Hispanic stroke survivors. The estimates showed the PWA had 71% (95% CI = −0.83, −0.20) lower income and 54% lower wealth (95% CI = −0.67, −0.35) compared to stroke survivors without aphasia. However, Black PWA had 31% (95% CI = −0.83, −0.20) lower income and 46% (95% CI = −0.67, −0.35) lower wealth than their counterparts without aphasia.

**Table 3:** Two-stage income and wealth regression estimates.

	**Dependent variable: income**						**Dependent variable: wealth**					
	**SE**		***t***	***p* > |*t*|**	**95% CI**		**SE**		***t***	***p* > |*t*|**	**95% CI**	
Intercept	**9.93**	0.20	49.07	0.00	9.53	10.33	**9.66**	0.24	41.07	0.00	9.20	10.12
Age ≥65	0.03	0.07	0.48	0.63	−0.10	0.16	**0.20**	0.08	2.55	0.01	0.05	0.34
Female	**−0.40**	0.07	−5.42	0.00	−0.54	−0.26	**−0.37**	0.08	−4.72	0.00	−0.52	−0.22
2+ Comorbidities	−0.24	0.17	−1.41	0.16	−0.56	0.09	−0.08	0.18	−0.44	0.66	−0.44	0.28
Black race	**−0.26**	0.08	−3.11	0.00	−0.42	−0.10	**−0.34**	0.10	−3.48	0.00	−0.54	−0.15
Hispanic ethnicity	**−0.55**	0.13	−4.24	0.00	−0.81	−0.30	**−0.53**	0.14	−3.74	0.00	−0.81	−0.25
Currently working	**0.27**	0.07	3.87	0.00	0.13	0.40	**0.38**	0.08	4.91	0.00	0.23	0.53
Private health insurance	**0.40**	0.07	5.61	0.00	0.26	0.54	**0.46**	0.08	5.97	0.00	0.31	0.61
Resides with ≥3 people	−0.14	0.09	−1.61	0.11	−0.32	0.03	**−0.18**	0.09	−1.94	0.05	−0.37	0.00
Midwest	**0.26**	0.12	2.26	0.02	0.03	0.49	**0.27**	0.12	2.27	0.02	0.04	0.51
West	0.10	0.09	1.13	0.26	−0.07	0.27	0.04	0.09	0.41	0.68	−0.14	0.21
South	−0.14	0.12	−1.19	0.23	−0.37	0.09	−0.24	0.12	−1.92	0.06	−0.48	0.00
Education beyond high school	**0.32**	0.08	4.06	0.00	0.17	0.48	**0.35**	0.08	4.13	0.00	0.18	0.51
High cost of healthcare	−0.04	0.07	−0.55	0.58	−0.19	0.11	−0.06	0.08	−0.78	0.43	−0.21	0.09
Aphasia	**−0.71**	0.23	−3.06	0.00	−1.17	−0.26	**−0.54**	0.23	−2.32	0.02	−1.00	−0.08
Black^*^aphasia	**−0.31**	0.26	−2.19	0.02	−0.83	−0.20	**−0.46**	0.26	−2.62	0.05	−0.67	−0.35
Hispanic^*^aphasia	−0.21	0.51	−0.40	0.69	−1.22	0.80	−0.27	0.47	−0.57	0.57	−1.20	0.66
	**Selection equation: income** > **0**						**Selection equation: wealth** > **0**					
Intercept	**−6.63**	0.13	−51.77	0.00	−6.88	−6.38	**−8.83**	0.04	−196.26	0.00	−8.91	−8.74
Age ≥65	**−0.47**	0.10	−4.85	0.00	−0.66	−0.28	**0.07**	0.02	3.05	0.00	0.02	0.11
Female	**−0.47**	0.04	−11.48	0.00	−0.56	−0.39	**0.04**	0.02	2.45	0.01	0.01	0.08
2+ Comorbidities	**−0.47**	0.10	−4.76	0.00	−0.66	−0.27	**0.07**	0.02	2.78	0.01	0.02	0.12
Black race	**−0.47**	0.13	−3.51	0.00	−0.73	−0.21	0.01	0.02	0.26	0.79	−0.04	0.06
Hispanic ethnicity	**−0.22**	0.11	−1.96	0.05	−0.44	0.00	−0.02	0.03	−0.63	0.53	−0.07	0.04
Currently working	**−0.20**	0.09	−2.28	0.02	−0.37	−0.03	−0.02	0.02	−0.81	0.42	−0.06	0.03
Private health insurance	**−0.24**	0.04	−5.47	0.00	−0.32	−0.15	0.00	0.02	0.16	0.87	−0.03	0.04
Resides with ≥3 people	**−0.21**	0.07	−2.78	0.01	−0.35	−0.06	**−0.05**	0.02	−2.69	0.01	−0.09	−0.01
Midwest	**−0.60**	0.09	−6.32	0.00	−0.78	−0.41	0.06	0.03	1.64	0.10	−0.01	0.12
West	**−0.52**	0.06	−8.33	0.00	−0.64	−0.40	0.00	0.03	0.02	0.99	−0.06	0.06
South	**−0.52**	0.06	−8.19	0.00	−0.64	−0.39	−0.01	0.03	−0.46	0.64	−0.08	0.05
Education beyond high school	**−0.32**	0.05	−6.48	0.00	−0.42	−0.22	0.00	0.02	0.18	0.86	−0.03	0.04
High cost of healthcare	**−0.45**	0.08	−5.69	0.00	−0.60	−0.29	**0.05**	0.02	2.36	0.02	0.01	0.08
Aphasia	0.00	0.11	0.05	0.96	−0.21	0.22	**−0.10**	0.03	−3.29	0.00	−0.15	−0.04
Black^*^aphasia	**1.00**	0.15	6.73	0.00	0.71	1.29	**−0.11**	0.03	−3.22	0.00	−0.17	−0.04
Hispanic^*^aphasia	0.12	0.13	0.89	0.37	−0.14	0.37	**−0.10**	0.05	−2.28	0.02	−0.19	−0.01

Reference group: Age (< 65), Sex (male), Comorbidities (0-1 comorbidities), Race (non-Black), Ethnicity (non-Hispanic), Household size (resides with 0-2 people), Region (Northeast), Education (high school degree or below), Healthcare cost (lower healthcare costs), Aphasia (no aphasia diagnosis).

Bold indicates significance at a 95% confidence interval.

## Discussion

The key finding of this study was that communication disability (aphasia) resulted in lower incomes and wealth. Additionally, Black PWA saw the greatest decreases in income and wealth. More specifically, the presence of aphasia resulted in a 21% decrease in income and a 7% decrease in wealth for PWA, yet Black PWA experienced decreases of 29% in income and 24% in wealth. We believe the distinction between income and wealth is important given that after the age of 65 income and wealth can differ substantially as individuals transition from earned income to fixed income. Consequently, the financing of healthcare for chronic conditions such as stroke becomes more difficult (Park and Stimpson, 2024). Furthermore, the impact of being Black and having a post-stroke communication disorder such as aphasia further magnifies the complexity of the income and wealth problem. We consider these collective issues in the context of known income and wealth disparities that exist among racial and ethnic minorities and individuals with communication issues such as aphasia.

### Race and income–wealth disparities among individuals with disabilities

Income and wealth disparities among individuals with disabilities have been previously reported (Jajtner et al., 2020). Households with adult members with a disability are two times more likely to live under the federal poverty line, have a higher liquid asset poverty rate, and earn more than $33,000 less than households without a disability (Prosperity Now, 2018). Individuals with disabilities frequently experience disadvantages related to employment, and subsequently are susceptible to economic vulnerability (Jajtner et al., 2020). Consequently, individuals with disability experience a “disability penalty” or disadvantage that further reduces their likelihood of employment and income and long-term wealth accumulation (Willson et al., 2024).

Income and wealth disparities are further impacted by racial and ethnic background. The National Disability Institute Report titled “Financial Inequality: Disability, Race, and Poverty in America” (Goodman et al., 2019) showed that racial and ethnic minorities and people with disabilities face barriers that limit their earning potential. The report notes a dynamic interaction between disability, race, and poverty (Goodman et al., 2019). More specifically, individuals from racial and ethnic minority groups are more likely to have a disability during working age compared to white Americans. Given the complex relationship between race and disability, many of these individuals experience higher healthcare costs while simultaneously being disadvantaged by their inability to work or earn equitable incomes (Goodman et al., 2019). The authors argue that disability is a cause and consequence of poverty while, at the same time, disability reinforces poverty (Goodman et al., 2019, p. 5). Even after adjusting for educational level, individuals from racial and ethnic minorities with disabilities are more likely to live in poverty than other disability groups. Ultimately, the authors conclude that race and disability are not completely different sources of disadvantage but are more likely to parallel one another (Goodman et al., 2019).

### Income–wealth disparities among individuals with communication disabilities

Whereas the intersection of race and disability translates into lower income and wealth, understanding the type of disability is equally important. Evidence suggests that individuals with communication disabilities in general experience economic disadvantage (unemployment and lower income) and are more commonly represented in low socioeconomic groups (Jagoe et al., 2021). Many workplaces require good communication, and those with disabilities are more frequently under-employed, unemployed, or lack ideal workplace integration (Garcia et al., 2002).

Regarding aphasia specifically, PWA often experience significant difficulty returning to work, and only a small percentage are successful in returning to their pre-aphasia jobs (Gilmore et al., 2022; Burfein et al., 2024; Pike et al., 2017). A recent review highlighted a range of factors that contribute to greater success in returning to work, including personal factors (aphasia severity, motivation, and fatigue), rehabilitation factors (access to vocational rehabilitation), and workplace factors (employer engagement in supported and job adaptations) (Gilmore et al., 2022). At the same time, PWA frequently experience reintegration challenges, which can be influenced by their perceived value in the workplace and the support they receive from others (Pike et al., 2017). Others are limited by linguistic profiling or “when judgments about the identity or subgroup based on auditory cues or speech characteristics” are made, which reduces their likelihood of re-engaging in the workforce (Caldwell et al., 2024; Baugh, 2000). Finally, it is unclear how individuals with communication disorders are aware that the Americans with Disabilities Act of 2008 expanded the definition of disability to include “speaking, hearing, and communicating,” and has therefore given them the protection of the law that precludes employers from asking about the presence of a communication disorder (Isetti and Eadie, 2016; Equal Employment Opportunity Commission, 2013). Clearly, some individuals are precluded from returning to work based on the severity of their aphasia, and therefore the presence of communication disorders is a major contributor to income and wealth inequities that many individuals with aphasia and other communication disorders experience.

## Limitations

Although this study provides valuable insights, it is subject to several limitations. First, the MEPS contains no information on the type of stroke or whether this was the first stroke or a recurrent stroke. Second, the MEPS does not contain any information about the type of acute stroke care received or any post-stroke rehabilitation services. Furthermore, these data do not allow for the identification of any morbidities, deficits, or physical symptoms other than aphasia that resulted from a stroke. Third, the racial and ethnic disparities in aphasia-related financial toxicity may be related to differences in the timing and/or quality of rehabilitation care received by different racial and ethnic groups; however, these data do not permit an assessment of these differences. Although the MEPS incorporates the Consumer Assessment of Healthcare Providers and Systems (CAHPS^®^) to measure the perceived quality of care, these subjective measures only cover the last 12 months and do not specifically address stroke care. Fourth, although the data identifies respondents who died during the panel, it does not specify the cause of death, which may affect the interpretation of the financial impacts related to stroke by resulting in censoring observations. However, a sensitivity analysis indicated that excluding deceased individuals from the study did not meaningfully alter the results. Fifth, this study relies on the MEPS data, which categorizes gender as either “male” or “female.” This binary classification does not capture non-binary or gender-diverse identities, potentially excluding populations that may experience unique healthcare challenges and financial toxicity. This limitation restricts the study's ability to generalize findings across the full spectrum of gender identities. Future research should consider incorporating more inclusive gender data to ensure a better understanding of stroke-related financial toxicity among all individuals. The lack of granularity in the data limits our ability to discern and discuss the nuanced differences that might exist within these broad categories, potentially obscuring specific factors that contribute to financial toxicity. Future studies should aim to collect and analyze data that distinguish across diverse communities. Additionally, incomplete and inaccurate sex, race, or ethnic data may limit the ability to understand the sources of disparities in healthcare costs, quality, and outcomes. Finally, the use of self-reported financial data may introduce reporting bias. While FE models control for unobserved heterogeneity, they cannot account for time-varying factors that might influence financial outcomes or fully investigate the specific mechanisms underlying these observed disparities.

## Conclusion

In conclusion, this study sheds light on the substantial financial burdens experienced by stroke survivors with aphasia, highlighting how communication disabilities potentially exacerbate the financial toxicity already present in stroke recovery. The findings underscore significant disparities in income and wealth between PWA and those without, particularly among racial and ethnic minorities, with Black PWA facing disproportionately greater financial challenges. These disparities emphasize the compounded disadvantage that individuals with both a communication disorder and a minority racial background endure, further complicating their ability to achieve financial stability. The study provides critical insight into the factors contributing to financial toxicity, such as reduced access to employment and the high costs of healthcare, which are particularly pronounced among those with aphasia.

These findings indicate a need for targeted interventions that address both the healthcare and financial needs of PWA. Policymakers and healthcare providers should consider the development of rehabilitation programs and job re-entry initiatives tailored to individuals with communication disabilities. Additionally, addressing racial disparities in healthcare access and post-stroke care is imperative to reduce the financial strain faced by minority groups. Future research should continue to explore the intersection of race, disability, and financial toxicity, with an emphasis on improving rehabilitation support, access to care, and economic outcomes for stroke survivors with aphasia. Such efforts will help ensure that all individuals, regardless of their communication abilities or racial background, have equitable opportunities for financial recovery and stability.

## Data Availability

Publicly available datasets were analyzed in this study. This study utilized Panel 23 Four-Year Longitudinal Data File (available at: https://meps.ahrq.gov/mepsweb/data_stats/download_data_files_results.jsp?cboDataYear=All&cboDataTypeY=1%2CHousehold+Full+Year+File&buttonYearandDataType=Search&cboPufNumber=All&SearchTitle=Longitudinal) and the HC 231, HC 222, HC 214, and HC 207 Medical Condition files (available at: https://meps.ahrq.gov/mepsweb/data_stats/download_data_files_results.jsp?cboDataYear=All&cboDataTypeY=1%2CHousehold+Full+Year+File&buttonYearandDataType=Search&cboPufNumber=All&SearchTitle=Medical+Conditions).
